# Ventral Hernia Repair Using Ventralex® ST Patch: A Single-Center Study of Clinical Outcomes and Complications

**DOI:** 10.7759/cureus.29341

**Published:** 2022-09-19

**Authors:** Birendra K Rajwade, Ravi V Patel, Yugal D Jain, Rajendra P Bhoge, Pradeep P Sharma

**Affiliations:** 1 General Surgery, Jehangir Hospital, Pune, IND

**Keywords:** ventral hernia, recurrence, hernia, umbilical, ventralex®-st patch

## Abstract

Background

Ventralex® ST (Bard Davol Inc, Warwick, RI) patch is a composite self-expanding and non-absorbable mesh used to reinforce ventral hernia repair. This study was conducted to assess the efficacy, post-operative clinical outcomes, the duration of operation, and complications deciding the post-operative duration/need of hospitalization in patients of small ventral hernia after their open surgical repair using Ventralex ST hernia patch.

Materials and methods

We included 36 patients diagnosed with a small (<2 cm defect size) ventral hernia who underwent open mesh repair following strict surgical methodology using Ventralex® ST hernia patch between September 2018 and April 2020. It was a prospective observational study. Clinically relevant characteristics, along with operative and post-operative data collected through direct interview, clinical examination, and a pretested proforma, were analyzed prospectively.

Results

Thirty-four patients (94.5%) were observed to have an operative time of fewer than 60 minutes, with an average duration of 30-40 minutes. Thirty-one patients (86%) were discharged within 48 hours of surgery, out of which 17 patients (47.2%) were discharged within 24 hours of surgery and 14 patients (38.8%) within 48 hours. Only five patients (13.88%) had a prolonged hospital stay for more than 48 hours due to post-operative complications. Three patients (8.33%) acquired post-surgical wound infection, whereas two (5.55%) developed seroma. Nevertheless, just one patient (2.7%) reportedly developed both infection and seroma after surgery. No cases of mesh infection or recurrences were noted.

Conclusion

This study demonstrates that open repair of small (<2 cm defect size) ventral hernia using the Ventralex® ST hernia patch can be an extremely safe and effective method. Furthermore, it has excellent clinical outcomes when meticulously used with an easily reproducible surgical technique, which requires less intra-operative time, has minimal post-operative complications and negligible recurrence rate, along with reduced post-op hospital stay (86% of patients being discharged within 48 hours).

## Introduction

Hernia can be defined as the abnormal protrusion of any organ (tissue) as a whole or part of it, out of its boundary through an anatomical or acquired weak spot or through a defect in its surrounding wall [1]. The hernias that occur through the anterior abdominal wall are ventral hernias. These hernias can be categorized as spontaneous or acquired by their anatomical location on the abdominal wall [2]. Based on the location, they can be further classified as epigastric, paraumbilical, umbilical, and or Spigelian hernias. The most common ventral abdominal wall hernia is the umbilical hernia. Surgery is the only definitive treatment for an adult with a hernia, usually reinforced with a prosthetic mesh. Small (<2 cm defect size) ventral hernias are usually operated on via an open approach because of lower cost and similar surgical outcomes as to laparoscopy [3,4]. Open mesh repair can be done using different mesh placement techniques, including onlay, sublay, inlay, or underlay placement. Most conventional single-layer polypropylene mesh used in open inguinal hernia are placed either onlay or sublay, but not intraperitoneally [5]. Intraperitoneal mesh placement using a very small (2-4 cm) incision may be very difficult as opposed to laparoscopy, where it is very easy. The device (Ventralex® ST [Bard Davol Inc, Warwick, RI] hernia patch) used in this study can be placed intraperitoneally through a very small incision. It has a polypropylene side that comes in contact with the peritoneum, whereas another side is polytetrafluoroethylene (PTFE) which provides a barrier for adhesion formation.

## Materials and methods

Study group

We included 36 patients with a small ventral hernia who underwent an open surgical repair using the Ventralex® ST hernia patch device between September 2018 and April 2020. Inclusion criteria were: (1) ventral hernia (reducible, partially reducible, and irreducible), including both uncomplicated and complicated (obstructed but not strangulated with perforation) hernia, (2) both male and female patients, (3) no restrictions on age, and (4) patients requiring hospitalization. In addition, the exclusion criteria were: (1) out-patient department (OPD) patients, (2) incidental and asymptomatic umbilical hernia in all patients of chronic liver disease (CLD), whether symptomatic or not and ascites, (3) recurrent hernias, (4) history of repair of ventral hernia along with inguinal hernia repair. The data were collected by directly interviewing and clinically examining the patients.

Surgical technique

**Figure 1:**
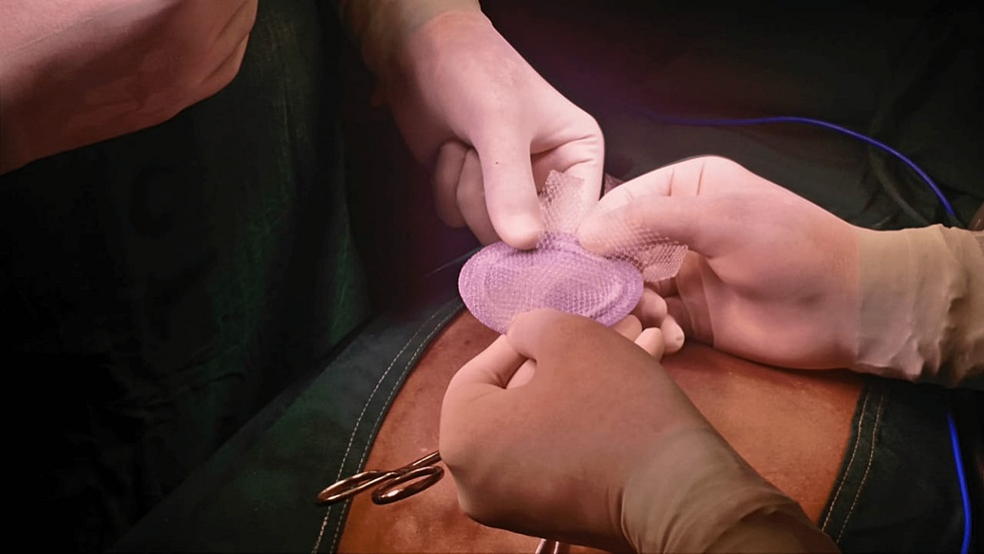
Ventralex® ST patch.

Ventralex® ST patch is a composite self-expanding and non-absorbable patch (Figure 1).

**Figure 2:**
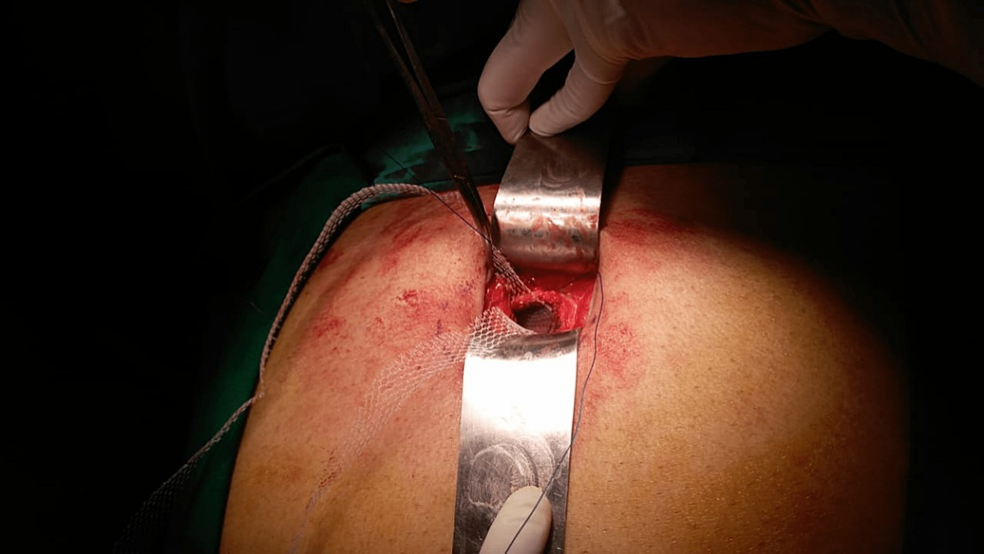
Inserted Ventralex® ST patch intra-abdominally with two flanges appearing outside.

It has a polypropylene (PP) side that remains in contact with the abdominal wall, encouraging tissue growth and integration. The visceral side is made of expanded polytetrafluoroethylene (ePTFE), facing the intraperitoneal space and providing a permanent barrier against adhesion formation. The main benefit of this technique is that fixation of the mesh is achieved principally by the intra-abdominal pressure that holds the prosthesis against the deep surface of the muscle, potentially improving tissue integration into the PP side of the mesh. All the patients were operated on using an open technique with a very small incision measuring 2-4 cm. After dissection of the hernial sac, contents were reduced, and a Ventralex® ST patch was introduced after activating its hydrophilic layer by soaking it in normal saline, keeping the flanges outside the abdomen to be sutured to the edges of the hernial defect (Figure 2).

**Figure 3:**
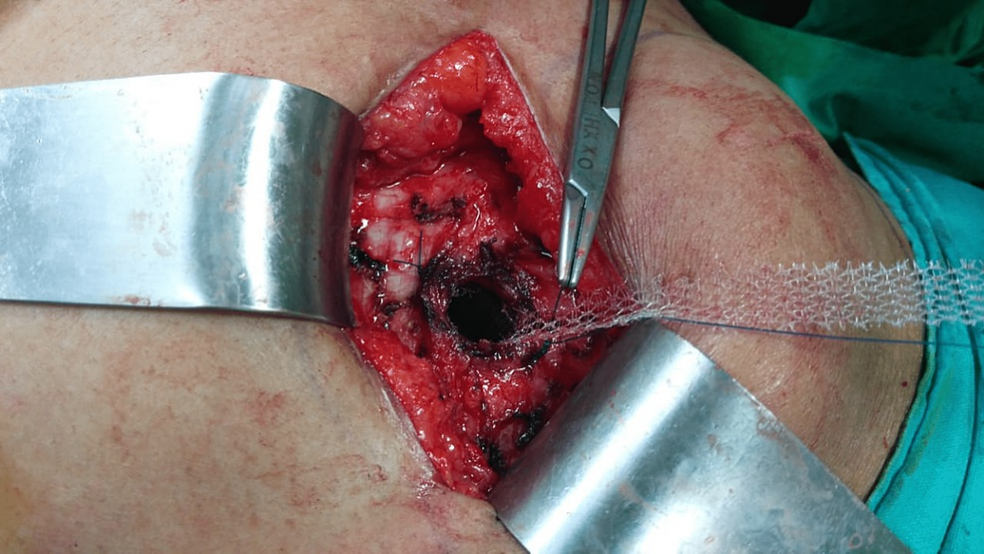
Flanges of patch being sutured at the edges of the hernia defect using non-absorbable polypropylene suture.

The patch was fixed using a PP suture to the anterior abdominal wall, and an extra length of flanges was trimmed. Post-operative outcomes and complications were observed and analyzed subsequently (Figure 3).

## Results

Out of 36 patients studied, the distribution of different types of ventral hernia is given in Table 1.

**Table 1:** Distribution of type of ventral hernia in study population.

S. No.	Type of hernia	Number of patients	Percentage
1	Umbilical hernia	17	47.22
2	Paraumbilical hernia	8	22.22
3	Epigastric hernia	6	16.66
4	Incisional hernia	4	11.11
5	Spigelian hernia	1	2.77
	Total	36	100%

There were 22 male (61.1%) and 14 female (38.9%) patients in the study group. As the sole clinical feature, 19 individuals (52.8%) presented with swelling. Rest 17 patients (47%) had associated symptoms like pain (13 patients, 36.1%) and pain and vomiting, both in four patients (11.1%). All patients had hernial defect sizes in the range of 1.5-3.5 cm. None had a size of more than 3.5 cm (Table 2).

**Table 2:** Distribution of the size of hernial defect in study population.

S. No.	Size of defect (in cm)	No. of patients	Percentage
1	<1.5	Nil	0
2	1.5-2.5	16	44.44
3	2.5-3.5	20	55.55
4	>3.5	Nil	0
	Total	36	100%

Thirty-four patients (94.5%) were observed to have an operative time of fewer than 60 minutes, with an average duration of 30-40 minutes. Thirty-one patients (86%) were discharged within 48 hours of surgery, out of which 17 patients (47.2%) were discharged within 24 hours of surgery and 14 patients (38.8%) in the next 24 hours. Only five patients (13.88%) had a prolonged hospital stay for more than 48 hours due to post-operative complications. In contrast, the majority of cases had no post-operative complications, and 16.6% of patients presented with minor post-operative complications. Three patients (8.33%) acquired post-surgical wound infection, whereas two patients (5.55%) developed seroma. Nevertheless, one patient (2.7%) reportedly developed both infection and seroma after surgery. No cases of mesh infection or recurrences were noted. We found a significant correlation between the duration of surgery and average hospital stay. Pearson correlation indicated that hospital stay was significantly (r=0.38, p<0.05) correlated with the duration of surgery (Table 3).

**Table 3:** Correlation of hospital stay with the duration of surgery.

		Duration of surgery
Hospital stay	Pearson's correlation	0.38
	P-value	0.022

## Discussion

Ventral anterior abdominal wall hernias include both spontaneous and, more commonly, incisional hernias. It is believed that more than 90% of adult ventral hernia cases are acquired diseases. Therefore, factors that contribute to the increase in intra-abdominal pressure (pregnancy, ascites, obstructive uropathy, chronic constipation), contractions of the abdominal wall muscles, and deterioration of the connective tissue predispose to this condition [6]. Due to the risk of incarceration and strangulation in adults with ventral hernias [7], elective surgical repair is recommended. Small (<2 cm) hernias are often successfully closed with primary tissue repairs. However, larger ones (>4 cm) have a recurrence rate of up to 30-40% when a tissue repair alone is performed. Hernia recurrence is distressing to patients and embarrassing to surgeons [8,9]. Thus, nowadays, tension-free repair using prosthetic mesh is preferred as they have a negligible recurrence rate. Tension-free mesh repair is the ideal hernia repair technique [10]. Small ventral hernia repairs can be done safely and securely under local or regional anesthesia with a tension-free mesh technique with low morbidity, negligible recurrence rate, and a high degree of patient satisfaction alleviating the risks of general anesthesia. It should be the procedure of choice for all such hernias [11]. 

Ventralex® ST hernia patch is an innovative mesh for open ventral hernia repair, which we have used in this study. In the present study, we observed umbilical hernias being the most common subtype of ventral hernia, comprising 70% of all the cases. In contrast, Hadi HI et al. observed a higher incidence of umbilical/paraumbilical hernia (86%) in their study, and Martin DF et al. found umbilical hernia the most common type comprising 60% of cases in their study [12,13]. In the present study, we observed that the majority of the cases presented were between 41-50 years of age with a mean age of 45.5 years, whereas Hadi HI et al. (mean age 52.4 years) and Agca B and Iscan Y (mean age 52.6 years) found a higher mean age at presentation [12,14]. Abdominal wall muscles and fascia lose their strength with increasing age, causing parietal weakness and microscopic tissue tears making them more prone to herniation. We observed that the ventral/umbilical hernia incidence was higher in males than in females. (M: F=1.5:1) which correlates with the data from Martin DF et al. (M: F=3.5:1) and Agca B and Iscan Y (M: F=1.2:1) [13,14]. The majority of the patients in our study presented with swelling as a primary complaint.

Similarly, Bharath PV et al. also found that most patients presented with swelling alone, without any associated symptoms [15]. In the present study, the majority of the patients had a hernial defect size between 2.5 and 3.5 cm. Hadi HI et al. found 1-1.5 cm size hernial defects in most patients in their study [12]. Agca B, Iscan Y, and Berrevoet F et al. found 2-4 cm and 1-3 cm hernial defects in most patients in their studies, respectively [14,16]. Umbilical hernias tend to have narrow necks compared to the size of the hernial sac and thus become more prone to obstruction or strangulation and require surgical correction to avoid such complications [17]. The mean operative time with Ventralex® ST patch was about 35 mins, which correlates with Hadi HI et al., Martin DF et al., and Agca B et al., who observed mean operative times of about 30 mins, 42 mins, and 35.9 mins, respectively [12,14]. So, we can say that Ventralex® ST patch repair can be done easily in a time of <60 minutes. In the present study, we found that the post-operative hospital stay was less than 24 hours in about 47.22% and assessed that most patients (86%) were discharged within 48 hours. We observed a lesser number of patients getting discharged within 24 hours (47.22%) as compared to the findings observed by Hadi HI et al. (84%) and Martin DF et al. (93%). We observed that 86% of all the patients were discharged within 48 hours, whereas the above authors found a slightly higher rate of discharge, 98% and 99%, respectively [12,13]. Thus we can say that after Ventralex® ST patch repair, the patient had less duration of hospitalization and was discharged early, which in turn was beneficial for the patient in view of the reduced overall cost of hospitalization. In this study, we found that most cases had no post-operative complications (83.33%). Rashid T et al. conducted a study on laparoscopic ventral hernia repair where they found mean hospital stay was 2.5 days, whereas, in our study, 86% of patients were discharged within 48 hours [18]. Thus, using Ventralex® ST patch repair, the mean hospital stay was also reduced. We found that only a small percentage of patients (16.6%) presented with minor post-operative complications, including seroma (5.5%), wound infections (8.33%), or both combined (2.7%). The lower incidence of seroma in this hernia repair can be attributed to the minimal flaps created during surgery. In open hernia repair, the flaps created are large compared to Ventralex® ST repair, where flaps created are minimal. Wang K and Berney CR also had similar findings, with 95% of patients having no post-operative complications [19]. In the present study, we also found no recurrence during 15 months of follow-up, whereas the recurrence rates observed by Hadi HI et al., Wang K, Berney CR, and Agca B, Iscan Y during their follow-up period of 12 months, 37.9 months, and 17 months, respectively are given in Table 4.

**Table 4:** Various studies showing rate of post-operative complications.

S. No.	Post-operative complications	Present study	Hadi HI et al.^ [12]^	Wang K and Berney CR^ [19]^	Agca B and Iscan Y​​​​​​​^ [14]^
1	Nil	83.33%	95.6%	95.6%	88.3%
2	Minor complications	16.6%	4.4%	4.4%	11.8%
3	Recurrences	Nil	Nil	Nil	Nil

Limitations

As our study was focused on clinical outcomes and complications of open small ventral hernia repair using Ventralex® ST patch, we have not compared laparoscopic ventral hernia repair or any other type of hernia repair using Ventralex® ST patch in our study, which can add further insights.

## Conclusions

Use of Ventralex® ST hernia patch in the repair of small ventral hernia by open method is a safe and efficient method, as it requires less duration of operation, has minimal post-operative complications, and has almost negligible recurrence rate during 15 months of follow-up. It has also reduced post-operative hospital stay and can be a cost-effective technique. It also reduces the chances of complications related to the use of general anesthesia.
